# PROBEAT: PRObiotic Bacterial gEnome Analysis Toolkit

**DOI:** 10.3390/cimb48080811

**Published:** 2026-08-11

**Authors:** Vesselin Baev

**Affiliations:** Department of Molecular Biology, Faculty of Biology, University of Plovdiv, Tzar Assen 24, 4000 Plovdiv, Bulgaria; baev@uni-plovdiv.bg

**Keywords:** bacterial genome analysis, illumina, ONT, probiotic gene markers, workflow

## Abstract

Whole-genome sequencing has become a central approach for investigating candidate probiotic bacterial isolates, but probiogenomic analysis often requires multiple independent tools, manual marker-gene searches and study-specific interpretation of heterogeneous outputs. PROBEAT (PRObiotic Bacterial gEnome Analysis Toolkit), a Dockerized workflow for the integrated and reproducible genome-based characterization of candidate probiotic isolates. PROBEAT combines standard bacterial genome analysis with specialized probiotic-oriented features, covering read processing, genome assembly and quality assessment, taxonomic confirmation, genome annotation, safety screening, functional and metabolic profiling, probiotic marker detection and automated visualization. A key feature of PROBEAT is a custom Probiotic Gene Markers database containing more than 400 gene aliases organized into 320 curated marker records and 32 functional categories. The workflow automatically converts raw sequencing data into a structured PDF booklet report that integrates safety, taxonomy, metabolism, functional annotation and probiotic-oriented data. PROBEAT reduces the technical burden of bacterial genome analysis and provides a reproducible framework for standardized genome-based assessment of probiotic potential, while supporting functional annotation rather than clinical claims.

## 1. Introduction

Probiotics are commonly defined as live microorganisms that, when administered in adequate amounts, provide a health benefit on the host [1]. However, the transition from a bacterial isolate to a credible probiotic candidate before wet-lab trials requires more than taxonomic identification or the detection of a few beneficial genes. Whole-genome sequencing has become a key approach for evaluating microbial identity, functional capacity, antimicrobial resistance, and other genomic features relevant to safety and application potential [2]. This is particularly important for lactic acid bacteria and other candidate probiotic taxa, where strain-level differences may determine whether an isolate is suitable for food, biotechnology, or biomedical use.

Modern bioinformatics offers thousands of specialized tools, databases, and workflows, yet this abundance creates a practical barrier for many microbiologists and applied researchers [3]. A typical bacterial genome analysis may require independent installation, configuration, execution, and interpretation of many different tools for read quality control, trimming, assembly, assembly quality assessment, taxonomy, annotation, antimicrobial resistance, virulence factors, plasmids, prophages, bacteriocins, secondary metabolites, carbohydrate-active enzymes, pathway reconstruction, and comparative genomics. Although these programs are individually powerful, their fragmented use often limits reproducibility and makes routine analysis difficult for non-specialist users. In this context, there remains a need for user-friendly systems that integrate all these bioinformatics tools and convert heterogeneous outputs into a coherent, automatically generated report.

Several pipelines have addressed parts of this problem for bacterial whole-genome sequencing. TORMES was developed as a user-friendly pipeline for Illumina-based bacterial WGS (Whole-genome sequencing) analysis, including quality filtering, de novo assembly, genome annotation, MLST (Multilocus sequence typing), antimicrobial resistance and virulence screening, and pangenome comparisons [4]. Bactopia provides a flexible Nextflow-based framework for bacterial genome analysis and comparative genomics, with strong emphasis on scalability and standardized processing [5]. ASA^3^P was introduced as an automatic, locally executable, and scalable pipeline for assembly, annotation, and higher-level analysis of closely related bacterial isolates [6]. Other bacterial WGS pipelines and related workflows further support automated analysis of bacterial genomes, including assembly, annotation, resistance, virulence, plasmid detection, SNP (Single-nucleotide polymorphism) analysis, and phylogenetic reconstruction. These systems have substantially advanced bacterial genomics by improving automation, scalability, and reproducibility.

Existing bacterial genome workflows efficiently perform assembly, annotation, taxonomic assignment, antimicrobial resistance and virulence screening, but few provide an end-to-end probiotic-oriented interpretation layer. Most general bacterial genome pipelines are not specifically designed to interlink genome data from the perspective of probiotic potential. They often provide limited integration of probiotic marker databases, and do not produce a unified probiotic-oriented report combining safety, function, metabolism, mobile elements, and candidate health-relevant traits.

PROBEAT (PRObiotic Bacterial gEnome Analysis Toolkit) integrates more than 30 established bioinformatics tools and custom probiotic-oriented modules into a single workflow for bacterial isolate genome analysis. It is designed to start from raw sequencing data and end with a complete, integrated genome characterization report. PROBEAT is implemented as a Dockerized Snakemake workflow with a Streamlit web interface, Redis-based worker execution, and automated PDF reporting. The workflow supports Illumina SE (single end) and PE (paired end) data, Oxford Nanopore, and hybrid analyses, and integrates quality control, read preprocessing, assembly, polishing, assembly assessment, taxonomic confirmation, genome annotation, antimicrobial resistance and virulence screening, plasmid and mobile element detection, bacteriocin and biosynthetic gene cluster prediction, carbohydrate-active enzyme annotation, KEGG/eggNOG-based functional profiling, gut–brain axis marker screening, probiotic marker detection, genome visualization, and final report generation.

Currently there are several tools focused on the probiotic bacterial analysis such as ProBioPred, iProbiotics, ProbML, Pato and metaProbiotics which represent valuable computational approaches for the rapid prioritization of candidate probiotic microorganisms [7,8,9,10,11]. ProBioPred applies genus-specific support vector machine models based on probiotic-associated, virulence and antimicrobial-resistance genes, whereas iProbiotics uses selected k-mer features extracted directly from whole-genome sequences. ProbML extends genome-based classification through multiple machine-learning models and supports the generation of customized classifiers. On the other hand, metaProbiotics is specifically designed to identify probiotic-like organisms from fragmented metagenomic bins.

Such existing probiotic prediction tools require an already assembled, and in some cases annotated, bacterial genome as their primary input. Consequently, read preprocessing, genome assembly, quality assessment and annotation must be performed independently before these tools can be applied. PROBEAT overcomes this limitation by accepting raw Illumina, ONT (Oxford Nanopore Technologies) or hybrid sequencing reads and integrating the complete analytical process. PROBEAT differs fundamentally from these systems because its primary objective is not to produce a single probiotic/non-probiotic probability. Instead, PROBEAT provides an end-to-end workflow that begins with raw Illumina, ONT, or hybrid sequencing reads and generates a comprehensive, traceable characterization of a bacterial isolate, integrated with a genome-wide landscape of probiotic-associated gene markers. This broader design makes it suitable for detailed genome characterization, and hypothesis generation before experimental validation, while remaining complementary to machine-learning tools intended for candidate prioritization.

A growing number of probiogenomic studies report candidate probiotic isolates through whole-genome sequencing and manually curated tables of genes linked to probiotic traits such as acid and bile tolerance, adhesion, stress resistance, antimicrobial activity, vitamin biosynthesis, and host interaction [12,13,14,15,16]. Although informative, this approach is labor-intensive, depends on study-specific marker selection and manual interpretation, and limits reproducibility across datasets. PROBEAT was developed to address this gap by automating the detection and functional organization of probiotic-associated genomic markers within a reproducible workflow, enabling standardized and scalable genome-based assessment of probiotic potential.

On top of a broad layer of established tools for bacterial genome analysis, PROBEAT adds a probiotic-focused interpretation layer. For this purpose, a literature-curated Probiotic Gene Markers database (PGMDB) was made containing more than 400 unique gene aliases organized into 320 curated probiotic marker records. These markers are linked to 32 functional categories such as gastrointestinal survival, adhesion and colonization, antimicrobial activity, vitamin biosynthesis, carbohydrate utilization, exopolysaccharide and biofilm formation, and gut–brain axis-related functions. Thus, PROBEAT does not only detect genomic features, but also organizes them into biologically interpretable probiotic traits of the analyzed isolate.

## 2. Implementation

PROBEAT was implemented as a modular, reproducible and containerized workflow for genome-based characterization of bacterial probiotic isolates. The system was designed to transform raw sequencing data into a structured probiotic-oriented report by combining established bacterial genome analysis tools with custom interpretation modules and curated marker databases. The workflow is orchestrated with Snakemake, which enables rule-based dependency tracking, automatic execution of intermediate steps, restartability after failed jobs, and transparent generation of per-sample outputs [17]. PROBEAT accepts four sequencing layouts: Illumina paired-end, Illumina single-end, Oxford Nanopore Technologies (ONT), and hybrid short-read/long-read datasets. Sample metadata are defined in a tabular configuration file, while global parameters, database locations, execution profiles and optional analyses are defined in a YAML (YAML Ain’t Markup Language) configuration file.

**Figure 1:**
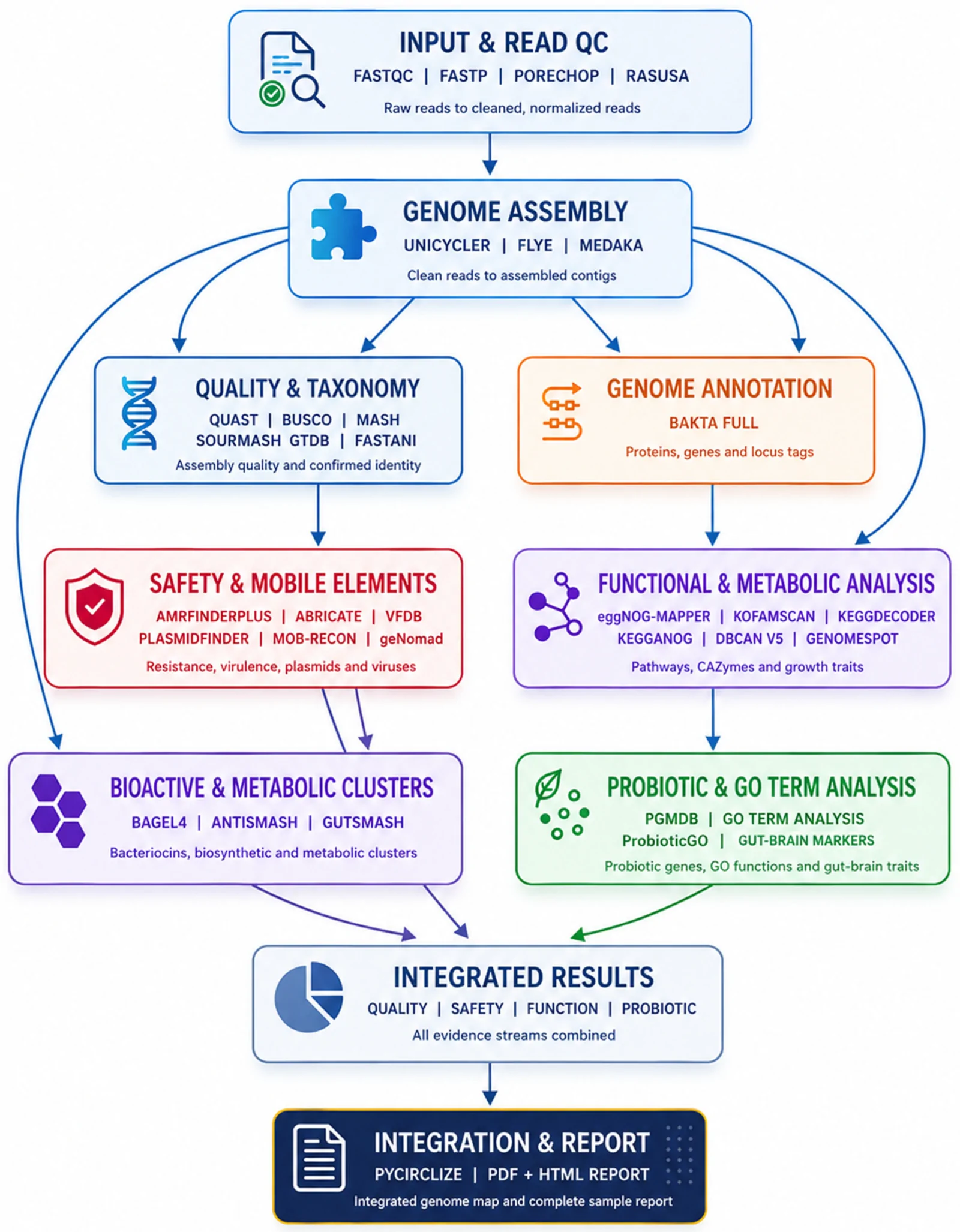
PROBEAT workflow summary diagram.

For each sample, the workflow creates a standardized directory structure containing raw-data quality control, preprocessed reads, genome assemblies, assembly quality metrics, taxonomic identification, genome annotation, functional profiles, safety-related screening, probiotic marker detection, visual summaries and final reports. The major analytical stages include: (i) read quality control and preprocessing; (ii) genome assembly and polishing; (iii) assembly quality assessment; (iv) taxonomic and comparative genome analysis; (v) structural and functional annotation; (vi) antimicrobial resistance, virulence, plasmid and mobile element screening; (vii) probiotic-oriented functional interpretation; and (viii) automated visualization and report generation. The complete workflow integrates more than 30 external bioinformatics tools and custom PROBEAT modules (Figure 1).

### 2.1. Workflow Architecture and Input Handling

PROBEAT was developed as a Snakemake-based directed acyclic graph in which each rule defines the required input files, output files, computational environment, execution parameters and log files. Input data are supplied as compressed or uncompressed FASTQ files (fastq or fastq.gz). Depending on the declared layout, PROBEAT selects the appropriate processing branch for paired-end Illumina, single-end Illumina, ONT-only, or hybrid assembly.

The principal input files are raw sequencing reads and a sample sheet defining sample identifiers and read paths. The final outputs are a per-sample PDF report, tabular summary files, intermediate analysis outputs and graphical summaries. PROBEAT also generates rule-specific logs, allowing failed or incomplete steps to be inspected without repeating the entire workflow. This architecture allows the workflow to remain both reproducible and extensible: new tools can be inserted as additional Snakemake rules, while existing tools can be updated by modifying the corresponding environment file, command block and output parser. Tools with their public repositories used in the workflow are listed in Table 1.

### 2.2. Read Quality Control and Preprocessing

For raw Illumina sequencing paired-end datasets, PROBEAT applies FastQC independently to the forward and reverse read files; for single-end datasets, the same rule is applied to the single input FASTQ file. The primary input is raw FASTQ data, and the output consists of sample-specific FastQC reports stored in the quality-control directory.

Illumina read trimming and filtering are performed with fastp v1.3.3 [18]. For paired-end data, fastp receives the raw R1 and R2 FASTQ files and produces trimmed paired FASTQ files together with HTML and JSON quality reports. For single-end data, fastp receives one raw FASTQ file and generates a trimmed single-end FASTQ file with the same report formats. These outputs are used both for downstream assembly and for quality documentation in the final report.

ONT reads are preprocessed with Porechop v0.2.4, which is used for adapter trimming of Nanopore reads (https://github.com/rrwick/porechop, accessed on 1 August 2026). In ONT-only analyses, the ONT FASTQ file is used as input; in hybrid analyses, the long-read file is processed through the same ONT preprocessing branch. The output is an adapter-trimmed ONT FASTQ file and an associated summary file. To standardize sequencing depth and reduce excessive computational load, PROBEAT applies Rasusa v2.1.0 to Illumina and ONT datasets [19]. Rasusa receives the trimmed FASTQ files and subsamples reads either to a target number of reads or to a target genome coverage. For ONT data, the default genome size parameter is set to 5 Mb and the default target coverage is 200×. The output consists of subsampled FASTQ files and a status table documenting the applied subsampling parameters. For illumina reads the files are downsampled up to 3M reads.

### 2.3. Genome Assembly, Polishing and Assembly Quality Assessment

Genome assembly is selected according to the sequencing layout. Illumina paired-end, Illumina single-end and hybrid assemblies are generated with Unicycler v0.5.1 [20]. For paired-end data, Unicycler receives the subsampled forward and reverse Illumina reads and produces a draft assembly in FASTA format. For single-end data, the single subsampled read file is passed to the assembler. For hybrid datasets, PROBEAT combines short-read input with preprocessed ONT reads, allowing Unicycler to use both sequencing technologies for assembly improvement. The principal output is assembly.fasta, which is used as the central genome file for downstream analyses.

ONT-only assemblies are produced with Flye v2.9.6, using the ONT-specific branch of the workflow [21]. Flye receives subsampled Nanopore reads and produces a long-read assembly. For ONT-only datasets, the Flye assembly is subsequently polished with Medaka v2.2 (https://github.com/nanoporetech/medaka, accessed on 1 August 2026). Medaka receives the ONT reads and the draft Flye assembly as input and produces a polished consensus assembly. The polished consensus sequence is then propagated to the downstream annotation, quality-control and screening modules.

Assembly quality is assessed with QUAST v5.3.0 [22]. QUAST receives the final assembly FASTA file and produces assembly statistics such as total length, number of contigs, N50, GC content and related metrics. Completeness is assessed with BUSCO v6.0.0 using the bacteria_odb10 lineage dataset [23]. BUSCO receives the assembly FASTA file and evaluates the presence, fragmentation or absence of conserved bacterial single-copy orthologs. The BUSCO output consists of a short summary file and detailed completeness reports, which are incorporated into the final report.

### 2.4. Taxonomic Assignment and Comparative Genome Analysis

PROBEAT combines rapid sketch-based screening with more detailed comparative genome analyses. Mash v2.3 is used for fast genome similarity estimation against RefSeq sketches [24]. The input is the assembled genome FASTA file, and the output is a table of top Mash hits, including approximate distances and matching reference genomes. In parallel for further verification, sourmash v4.x is used to generate genome sketches and search against the GTDB (Genome Taxonomy Database) representative genome database, configured as GTDB rs220 k-mer 31 [25]. The workflow produces sourmash gather results and lineage annotations that support taxonomic consistency checks.

FastANI v1.34 is used for average nucleotide identity estimation against selected reference genomes [26]. PROBEAT selects up to 15 reference genomes from the sourmash taxa results, downloads the corresponding sequences where available from NCBI, and calculates ANI (Average nucleotide identity) values between the query assembly and the reference set. The output is an ANI table and a comparative heatmap, supporting strain-level interpretation. Species-level assignment was considered consistent when the query genome showed ≥95% ANI to representative reference genomes, together with sufficient genome alignment coverage and concordant sketch-based taxonomic evidence.

### 2.5. Structural and Functional Genome Annotation

Genome annotation is performed with Bakta v1.12.0 using the Bakta full database v6.0 [27]. Bakta receives the assembled genome FASTA file and produces a comprehensive annotation package including GFF3, FAA, FNA, TSV, TXT and graphical outputs. The protein FASTA output from Bakta is used as a central input for several downstream functional modules, including eggNOG-mapper, KOfamScan and dbCAN.

Functional annotation is performed with eggNOG-mapper v2.1.x using the eggNOG v7 database [28]. The input is the Bakta-derived protein FASTA file, and the output is an annotation table containing orthology assignments, functional descriptions, COG (Clusters of Orthologous Groups) categories, KEGG orthology identifiers, enzyme commission numbers and Gene Ontology terms. PROBEAT parses these outputs to generate transport-related summaries, enzyme class summaries, COG visualizations and downstream probiotic-oriented analysis.

KOfamScan v1.3.0 is used for KEGG orthology assignment based on profile hidden Markov models from the KOfam database [29]. The input is the predicted protein FASTA file, and the output is a tabular KO (KEGG Orthology) assignment file. KEGGDecoder v1.3 is then used to infer pathway completeness and metabolic potential from KOfamScan results (https://speeding-up-science-workshops.github.io/autodocs/kegg-decoder/, accessed on 1 August 2026). KEGGDecoder outputs pathway summary tables and pathway visualization files. In addition, PROBEAT integrates KEGGaNOG for KEGG pathway extraction from eggNOG-mapper annotations. KEGGaNOG receives the eggNOG annotation file and produces KEGG pathway tables and graphical pathway summaries.

Carbohydrate-active enzymes are identified with dbCAN v5.2.9 and the dbCAN v5 database [30]. The dbCAN module receives the predicted protein FASTA file and the genome assembly and searches for CAZyme families, carbohydrate gene clusters and substrate predictions. The resulting CAZyme overview tables and substrate-related outputs are used to infer carbohydrate utilization potential, which is especially relevant for probiotic strains with prebiotic substrate metabolism.

### 2.6. Antimicrobial Resistance, Virulence, Plasmids and Mobile Genetic Elements

Antimicrobial resistance genes are screened with AMRFinderPlus v4.2.7 using the AMRFinderPlus database release 2026-05-15.1 [31]. The input is the assembled genome sequence, and the output is a tab-delimited table of detected antimicrobial resistance genes, point mutations where applicable, gene symbols, sequence coverage, identity and resistance class information.

PROBEAT also uses ABRicate v1.0.1 for additional sequence-based screening against several databases (ABRicate software repository). In the workflow, ABRicate is applied to the assembled genome to screen against CARD (Comprehensive Antibiotic Resistance Database) and MEGARes for antimicrobial resistance-associated sequences, against PlasmidFinder for plasmid replicon detection, and against VFDB (Virulence Factor Database) for virulence factor detection [32,33]. The outputs are database-specific TSV files that are parsed into the final report and integrated into the safety interpretation layer.

Plasmid and mobile element analyses are further extended with MOB-suite v3.1.9 and geNomad. MOB-suite receives the genome assembly and produces contig-level and plasmid-typing reports, including mobility and replicon-related information where available [34]. geNomad is used to identify virus- and plasmid-associated sequences in the assembled genome [35]. Its outputs include plasmid and virus summary tables, which help to evaluate the mobile genetic element content of candidate probiotic isolates.

### 2.7. Bacteriocins, Biosynthetic Gene Clusters and Specialized Probiotic-Associated Functions

Bacteriocin gene clusters are predicted with BAGEL4, installed in the PROBEAT environment [36]. BAGEL4 receives the genome assembly and annotation-derived information and produces bacteriocin cluster summaries and graphical outputs. In cases where the standard summary is empty, PROBEAT includes a fallback detection strategy to improve the extraction of bacteriocin-related hits for report generation.

Secondary metabolite biosynthetic gene clusters are predicted with antiSMASH v8.0.4 using bacterial settings and Prodigal-based gene prediction [37]. The input is the genome assembly, and the outputs include the antiSMASH HTML report, region files and tabular summaries of predicted biosynthetic gene clusters. To complement this analysis, PROBEAT integrates gutSMASH for the prediction of gut microbiome-relevant specialized metabolic pathways [38]. gutSMASH receives the genome assembly and returns pathway or status outputs that are summarized in the final report.

GenomeSPOT is used as an additional phenotypic prediction module for microbial growth-associated traits (GenomeSPOT preprint). The tool receives genome-derived sequence information and produces predictions related to organism-level ecological and physiological properties. In PROBEAT, these predictions are treated as supportive descriptors rather than definitive phenotype assignments.

### 2.8. Probiotic Marker Database and Custom Interpretation Modules

An important component of PROBEAT is the custom Probiotic Gene Markers database (PGMDB), developed for this study. The database currently contains more than 400 unique gene aliases organized into 320 curated probiotic marker records within 32 functional categories. These categories include gastrointestinal survival, acid stress, bile resistance, adhesion and colonization, antimicrobial activity, bacteriocin production, vitamin biosynthesis, carbohydrate utilization, exopolysaccharide production, biofilm formation, antioxidant activity, short-chain fatty acid production, polyamine metabolism, tryptophan metabolism and gut–brain axis-related functions.

The PGMDB module receives Bakta annotation tables, eggNOG-mapper functional annotations and the PGMDB marker table as input. It searches for matches between predicted genes, functional annotations and curated probiotic gene markers. The outputs are a marker-hit table and a summary table reporting detected probiotic-associated genes and their functional categories. These outputs are further processed by PROBEAT custom plotting scripts to generate functional potential visualizations. Marker hits were classified by evidence level according to the source of the match: direct Bakta gene/product annotation, eggNOG-derived functional annotation, or pathway-level marker inference. Hits were interpreted as genomic potential and were not treated as evidence of gene expression or phenotype.

A separate gut–brain axis marker module uses a curated gut–brain axis marker database developed for PROBEAT. It receives Bakta annotations, eggNOG-mapper annotations and the marker database as input and produces a table of detected genes associated with neurotransmitter metabolism, amino acid metabolism, polyamine production and other gut–brain axis-related functions.

Gene Ontology-based functional profiling is performed with the custom GOProbioticProfiler module. The module uses eggNOG-derived GO (Gene Ontology) terms, PGMDB hits, GO term definitions from go-basic.obo, and curated probiotic GO Slim mappings. GO processing and enrichment-related operations are supported by GOATOOLS [39]. The outputs include GO enrichment summaries, probiotic GO Slim profiles, bubble plots and general/probiotic-specific GO visualizations.

It is important to note that these modules are intended to support the functional detection and annotation of genomic features associated with probiotic potential rather than make clinical claims, predict therapeutic efficacy, or replace wet-lab validation.

### 2.9. Visualization, Reporting and Deployment

PROBEAT generates multiple visualization layers from the outputs of the analytical modules. Genome-level visualization is performed with pyCirclize, which receives genome annotation, antimicrobial resistance hits, plasmid and virulence screening results, CAZyme predictions, probiotic marker hits, bacteriocin predictions and biosynthetic gene cluster information. The output is a circular genome map in PNG and SVG formats. MultiQC v1.35 is used to aggregate quality-control and workflow-level information into a single report [40].

The final reporting step combines workflow outputs into a per-sample integrated report. This report includes sequencing and preprocessing summaries, assembly statistics, taxonomic evidence, annotation statistics, AMR (Antimicrobial resistance) and virulence screening, plasmid and mobile element results, bacteriocin and biosynthetic gene cluster predictions, carbohydrate metabolism, functional annotation, probiotic marker detection, gut–brain axis-related markers, GO-based summaries and genome visualizations. The main report is rendered as PDF, providing a complete analysis booklet (more than 40 pages) suitable for biological interpretation and downstream manuscript preparation.

For portability and reproducibility, PROBEAT is distributed as a Dockerized application. The container is built on a Micromamba-based image and installs the required Conda environments, Python 3.14 dependencies, external tools and custom scripts. The Docker Compose deployment includes a Streamlit web interface, a Redis service for job management, a background worker for workflow execution, and persistent volumes for uploaded reads, databases, intermediate files, final results, logs and environment caches. This deployment model allows users to run PROBEAT as a user-friendly web-accessible workflow while preserving the underlying reproducibility of the Snakemake execution layer.

## 3. Results

### 3.1. PROBEAT Workflow Overview

The workflow diagram (Figure 1) highlights an important design principle of PROBEAT: the system does not follow a strictly linear pipeline after genome assembly. Instead, the assembled genome is distributed to several parallel analytical branches. Quality and taxonomy are assessed using tools such as QUAST, BUSCO, Mash, sourmash and FastANI. Genome annotation is performed with Bakta, and the annotated genome becomes the basis for functional, metabolic and probiotic-oriented interpretation. Safety and mobile element screening are performed using AMRFinderPlus, ABRicate, VFDB, PlasmidFinder, MOB-recon and geNomad. In parallel, bioactive and metabolic clusters are detected using BAGEL4, antiSMASH and gutSMASH, while functional and metabolic potential is evaluated using eggNOG-mapper, KOfamScan, KEGGDecoder, KEGGaNOG, dbCAN and GenomeSPOT. The final stages merge independent evidence streams into integrated results. PROBEAT combines quality, safety, functional and probiotic-oriented evidence and generates both visual summaries and a complete PDF report. The report includes an integrated circular genome map produced with pyCirclize and a structured report booklet containing the results of each analysis module.

### 3.2. Graphical User Interface

**Figure 2:**
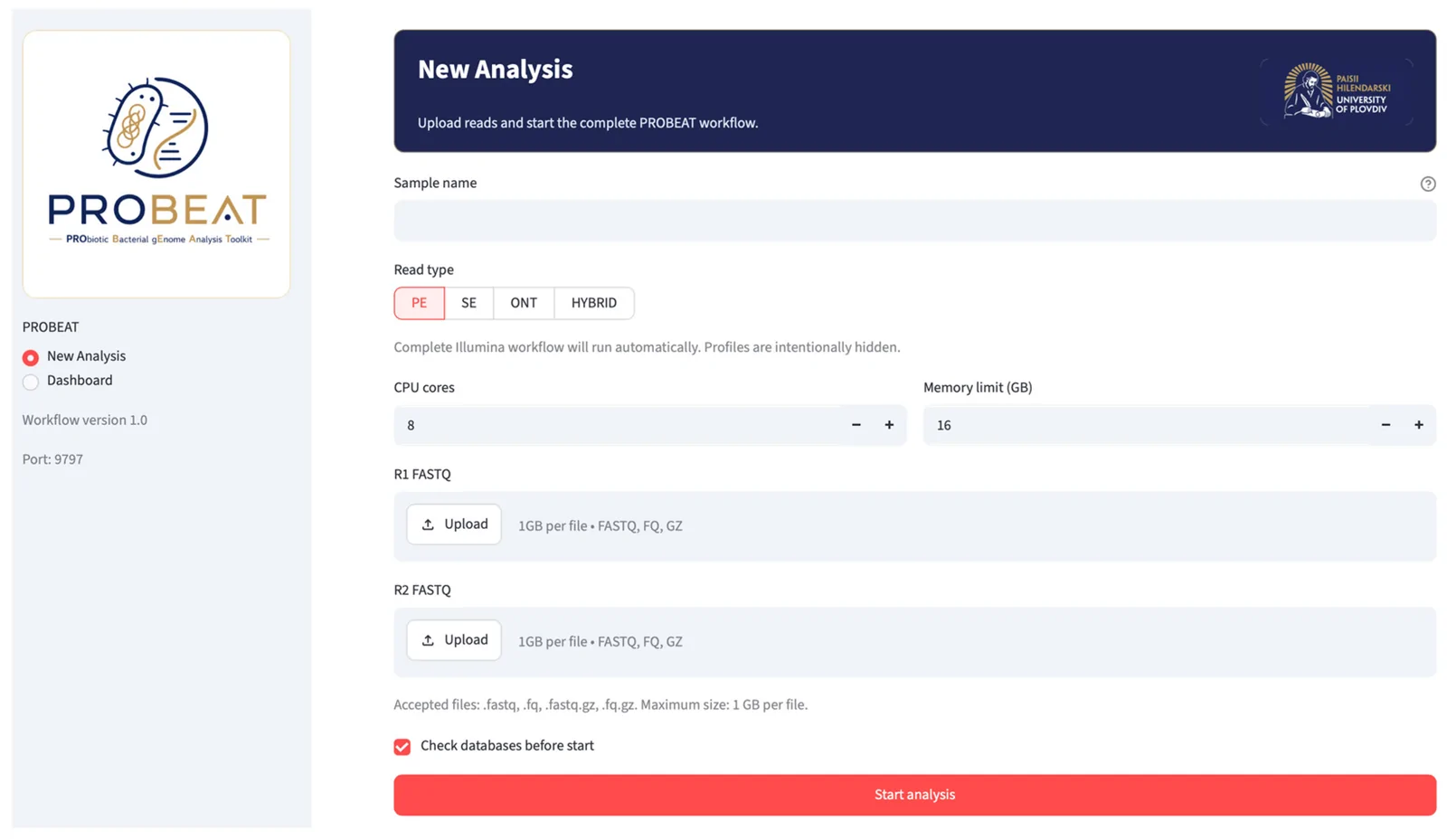
PROBEAT user web-interface.

The web interface of PROBEAT is shown in Figure 2. The “New Analysis” page provides a simplified entry point for users who do not want to interact directly with command-line tools or workflow configuration files. The user defines a sample name, selects the read type and uploads the corresponding sequencing files. The supported read types are paired-end Illumina, single-end Illumina, ONT and hybrid datasets.

The interface also allows the user to define computational resources, including CPU cores and memory limit. For paired-end Illumina datasets, the interface requests R1 and R2 FASTQ files; for ONT and single-end analyses, the required input fields are adjusted accordingly. The interface hides unnecessary workflow profiles from the user and exposes only the essential parameters required to start the full analysis. This design supports the main objective of PROBEAT: to provide a single pipeline from raw sequencing data to a complete bacterial probiotic isolate genome report.

The interface includes an optional database check before starting the analysis. This step reduces execution errors caused by missing reference databases and improves reproducibility by ensuring that required resources are available before the workflow is launched. Once the user starts the analysis, the uploaded files are passed to the background workflow execution layer.

### 3.3. Dashboard and Workflow Monitoring

**Figure 3:**
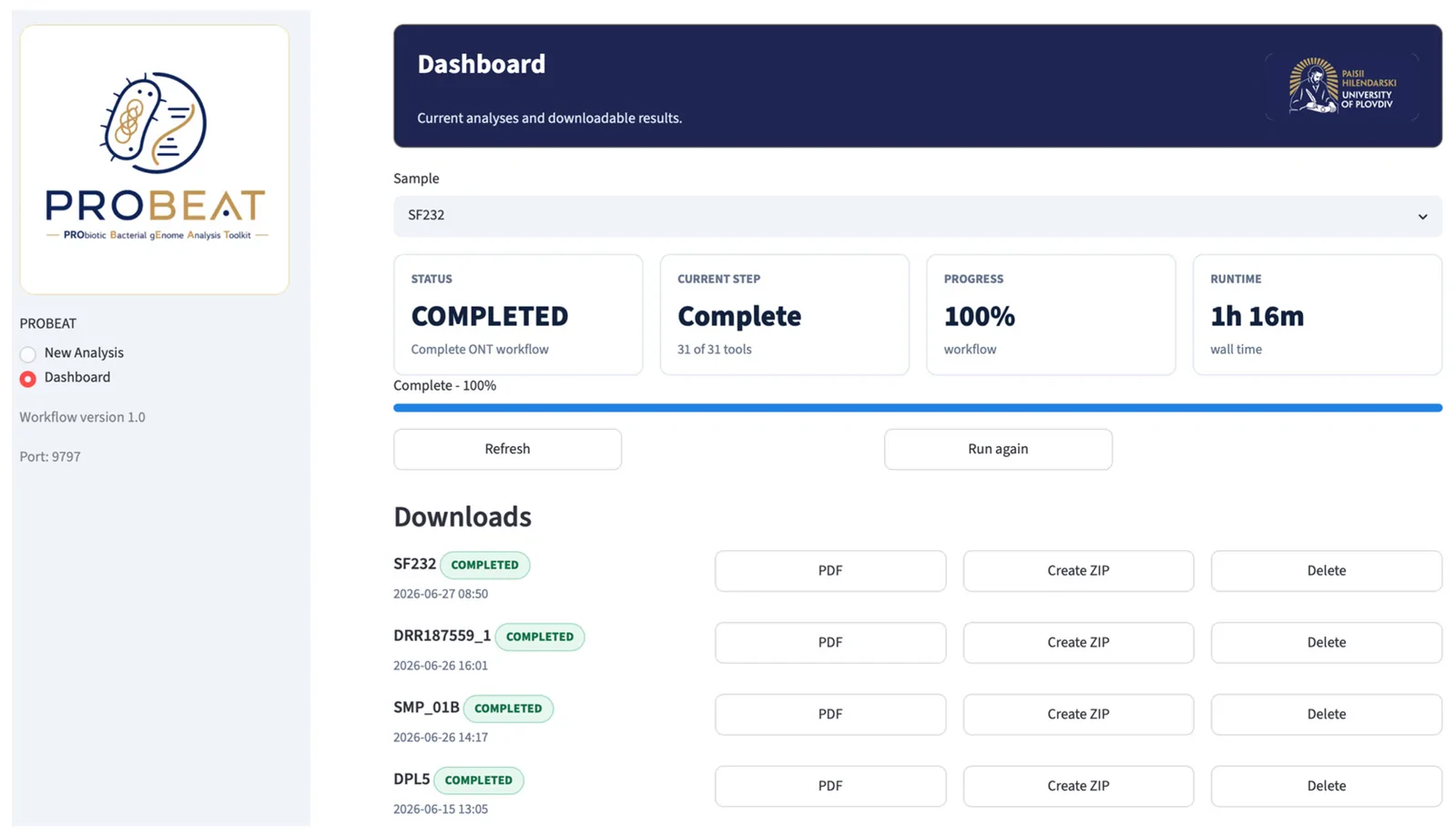
PROBEAT dashboard and results page.

The dashboard interface is shown in Figure 3. After a workflow has been launched, PROBEAT provides a simple monitoring panel showing the current sample, workflow status, current step, progress percentage and runtime. In a completed workflow, the dashboard reports the final status, total progress and number of successfully completed tools.

The dashboard also provides direct access to downloadable results. For completed analyses, users can download the generated PDF booklet report, create a ZIP archive of the complete result directory, rerun an analysis or delete previous results. This makes the platform suitable for iterative analyses of multiple bacterial isolates, where each sample can be tracked independently and retrieved as a self-contained result package.

### 3.4. Organization of the Generated Report

**Figure 4:**
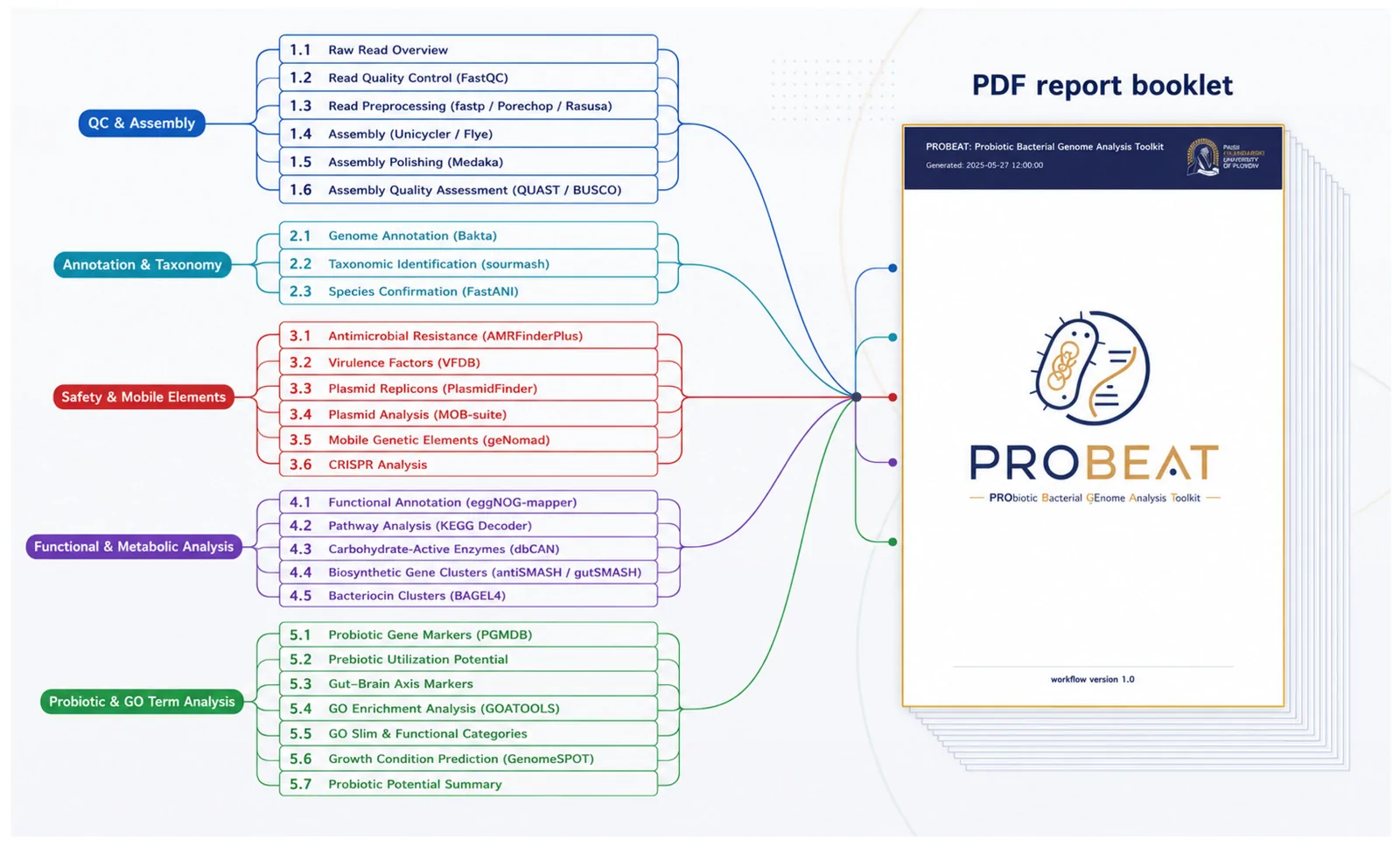
PROBEAT PDF booklet report. (For more details see full booklet st https://github.com/vebaev/PROBEAT/blob/master/docs/examples/SRR30409709_sample_booklet_report.pdf, accessed on 1 August 2026).

The PROBEAT report is organized as a multi-layered document that combines general genome analysis, safety assessment, metabolic reconstruction and probiotic-oriented interpretation (Figure 4). Each section corresponds either to a specific workflow stage, an external bioinformatics tool, or a custom PROBEAT module. This structure allows the user to move from high-level conclusions to traceable tool-level evidence. The report therefore functions both as a readable biological summary and as a technical record of all major output files produced by the workflow. A sample report for the public available data SRR30409709 (ONT data from Levilactobacillus brevis CHEE98) can be downloaded and explored from the GitHub repository at: https://github.com/vebaev/PROBEAT/blob/master/docs/examples/SRR30409709_sample_booklet_report.pdf, accessed on 1 August 2026.

**Overview and workflow**. This report section provides the first high-level summary of the analysis. It presents core genome and workflow metrics, including read number, assembly size, number of contigs, GC content, N50 and predicted species. It also includes the graphical workflow overview, which shows how the sample progresses from raw reads to integrated results and final report generation.

**Per-sample summary**. This section condenses the most important results into a compact sample-level overview. It summarizes quality control, assembly statistics, taxonomic identification, annotation output, plasmid and mobile element screening, antimicrobial resistance and virulence results, functional annotation, probiotic marker detection, gut–brain axis markers and GO-based probiotic categories. It is intended as the main quick-inspection page for users who need an immediate overview before examining individual modules in detail.

**Assembly report**. The assembly part reports contig-level statistics, including contig identifiers, contig length, coverage, circularity, repeat status, multiplicity and assembly grouping where available. Depending on the sequencing mode, this section reflects the output of the relevant assembly branch, such as Flye/Medaka for ONT data or Unicycler for Illumina and hybrid datasets. This section provides the structural genome foundation for all downstream annotation, screening and interpretation steps.

**Bakta annotation report**. The annotation section summarizes genome annotation generated with Bakta. It reports sequence length, contig count, GC content, N50, coding density, transfer RNAs (tRNAs), ribosomal RNAs (rRNAs), and transfer-messenger RNAs (tmRNAs), ncRNAs (non coding RNAs), CDSs (coding sequences), pseudogenes, hypothetical proteins, CRISPR (Clustered regularly interspaced short palindromic repeats ) arrays, gaps and predicted replication origins where available. This section also documents the Bakta software and database version, providing traceability for genome feature prediction.

**Integrated bacterial genome map**. This part provides a circular genome visualization integrating multiple evidence layers into one figure. The map combines forward and reverse CDS tracks, AMR or virulence-associated hits, antiSMASH regions, BAGEL4 bacteriocin/RiPP regions, dbCAN CAZyme hits, PGMDB probiotic gene markers, GC content and GC skew. This visualization allows users to inspect the spatial distribution of functionally important loci across the assembled genome.

**FastANI species confirmation**. This section reports genome-level species confirmation using average nucleotide identity. It compares the assembled genome against selected reference genomes and presents ANI values as a heatmap or tabular summary. The section helps validate whether the taxonomic assignment inferred from sketch-based methods and genome databases is consistent with genome-wide nucleotide identity.

**geNomad plasmid and virus summary**. This section reports plasmid- and virus-associated sequences predicted by geNomad. It includes contig identifiers, sequence length, topology, classification score, FDR (False discovery rate) where available, hallmark genes and marker enrichment. The section is important for assessing the potential presence of mobile genetic elements that may influence strain safety, genome plasticity or horizontal gene transfer risk.

**MOB-recon contig classification**. This section reports plasmid contig classification and reconstructed plasmid groups from MOB-suite. It includes plasmid-associated contigs, replicon types, relaxases, mobility predictions, nearest neighbors and predicted host range where available. Together with geNomad, this module provides complementary plasmid and mobility evidence.

**AMRFinderPlus**. This section reports antimicrobial resistance genes detected by AMRFinderPlus. The output provides a curated AMR screening layer and includes resistance gene names, sequence identity, coverage, resistance class and additional annotations where present. This section is central for safety-oriented genome screening of candidate probiotic isolates.

**VFDB hits**. This section reports virulence-factor database matches. It includes contig, gene name, product description, coverage and sequence identity. The section allows users to evaluate whether the analyzed genome contains loci with similarity to known virulence-associated genes and to inspect the strength of each match.

**ABRicate CARD and MEGARes hits**. These sections report additional AMR-associated matches detected using CARD and MEGARes databases. They provide a complementary sequence-similarity screening layer that may identify resistance-related homologs not reported by AMRFinderPlus. By including multiple AMR resources, PROBEAT allows users to compare conservative curated gene calls with broader database-driven similarity evidence.

**BAGEL4 bacteriocin and RiPP clusters**. This section reports bacteriocin and RiPP-associated areas of interest. It includes genomic coordinates, cluster class, predicted sequence type and graphical cluster representations when available. This module is particularly relevant for probiotic candidates because bacteriocin production may contribute to competitive exclusion and antimicrobial activity.

**antiSMASH biosynthetic gene clusters**. This section reports predicted biosynthetic gene clusters and their product classes. It includes region coordinates, cluster length, predicted product type and gene counts. The section helps identify specialized metabolic potential such as NRPS (Non-ribosomal peptide synthetase), PKS (Polyketide synthase), terpene, lanthipeptide or other biosynthetic regions that may contribute to strain-specific bioactivity.

**dbCAN CAZyme summary**. This section summarizes carbohydrate-active enzyme families detected in the genome. It reports glycoside hydrolases, glycosyltransferases, polysaccharide lyases, carbohydrate esterases, auxiliary activity enzymes and carbohydrate-binding modules. These results provide the primary enzymatic evidence for carbohydrate degradation, polysaccharide modification, cell wall metabolism and potential prebiotic substrate utilization.

**dbCAN prebiotic carbohydrate utilization**. This section extends the standard dbCAN output by linking CAZyme family hits to a custom PROBEAT probiotic database layer. In addition to reporting CAZy families, the module uses PGMDB-associated prebiotic carbohydrate mappings to connect detected CAZymes with probiotic-relevant substrate groups such as fructooligosaccharides, inulin-type fructans, galactooligosaccharides, lactose-derived oligosaccharides, xylooligosaccharides, arabinoxylan, arabinan, beta-glucans, starch-related substrates, pectin-related substrates and cello-oligosaccharides. The section reports substrate groups, detected CAZy families, corresponding Bakta locus tags and typical enzymatic functions. This converts raw CAZyme annotations into a biologically interpretable summary of putative prebiotic carbohydrate utilization potential.

**dbCAN CGC substrate predictions and synteny maps**. These sections report carbohydrate gene clusters with predicted substrates and visual synteny relationships to reference polysaccharide utilization loci. The substrate prediction table lists CGC (Carbohydrate gene cluster) identifiers, genomic coordinates, length, number of CAZymes, reference PUL (Polysaccharide utilization locus) identifiers, predicted substrates and similarity scores. The synteny maps provide a visual comparison between predicted gene clusters and reference loci, supporting interpretation of whether carbohydrate utilization potential is encoded in coherent genomic regions.

**PGMDB probiotic gene summary**. This section summarizes probiotic marker hits detected using the custom Probiotic Gene Markers Database. The summary section reports total marker hits, unique marker counts and functional category-level representation.

**PGMDB probiotic gene coverage**. This section visualizes the extent to which probiotic marker categories are represented in the analyzed genome. It reports category coverage, number of unique genes, number of unique hits and evidence strength. The visualization groups marker categories into broader probiotic-relevant domains, such as core survival, host interaction and metabolic benefit. This allows users to distinguish strongly represented categories from partial or weakly supported categories.

**PGMDB probiotic genes**. This detailed section provides the traceable evidence behind the PGMDB summary. For each detected probiotic function, it reports the marker gene or gene-alias group, matching Bakta locus tags and annotation descriptions. The database structure is designed to handle gene synonyms and pathway-level marker groups, because probiotic traits are often associated with operons, paralogous gene families or functionally related enzyme sets rather than single unique gene names. PROBEAT first searches for marker evidence in Bakta annotations and then uses eggNOG-derived functional annotations when a marker is not found directly in the primary annotation layer. This two-stage strategy increases marker recovery while preserving traceability to genome features. The section should be interpreted as evidence of genomic potential; the presence of a marker does not by itself demonstrate expression, metabolite production or probiotic activity.

**Gut–Brain axis markers**. This section reports genes associated with gut–brain axis-related metabolic traits. It includes detected markers, Bakta loci, KO or EC hints, marker logic and interpretation. The section covers pathways and traits such as GABA-related acid tolerance, GABA shunt, tryptophan precursor pools, aromatic amino acid metabolism, agmatine, putrescine, spermidine, cadaverine, vitamin biosynthesis, SCFA-related routes, exopolysaccharide production, mucosal persistence, oxidative stress resistance and bile survival. Interpretations are reported cautiously as genome-based potential rather than confirmed host effects.

**KEGGDecoder functional completeness**. This section summarizes pathway completeness based on KOfamScan-derived KO annotations. It highlights functions relevant to probiotic genome interpretation, including vitamin biosynthesis, central carbohydrate metabolism, fermentation, oxidative stress response, amino acid biosynthesis, peptidoglycan biosynthesis, exopolysaccharide or capsule-related functions, carbohydrate transport, and acid or bile tolerance. These results provide a pathway-level view of metabolic capacity.

**KEGGaNOG pathway completeness**. This section reports pathway completeness inferred from eggNOG-mapper annotations. It provides an additional orthology-based view of metabolic potential and complements the KOfamScan/KEGGDecoder branch. By using eggNOG-derived annotations, the report can summarize broader pathway signals that may not be fully captured by strict KO-profile matching alone.

**Nutrient uptake potential**. This section summarizes transporter families extracted from eggNOG-mapper annotations, with emphasis on probiotic relevance. It includes carbohydrate uptake systems, oligosaccharide transporters, peptide uptake systems, amino acid and polyamine transporters, osmotic stress-related transporters, multidrug or bile-associated efflux systems and ion homeostasis transporters. This helps connect genome annotation with potential nutrient acquisition, gut adaptation and stress tolerance.

**EC enzyme classification**. This section organizes predicted enzymes according to Enzyme Commission classes and functional subcategories. This provides an enzyme-centric view of metabolic capacity.

**COG functional categories**. This section provides a radar-style overview of COG functional categories derived from eggNOG-mapper annotations. It summarizes the distribution of broad functional classes such as carbohydrate transport and metabolism, amino acid metabolism, translation, transcription, replication and repair, cell wall biogenesis, secondary metabolism and inorganic ion transport. The plot gives a compact functional fingerprint of the analyzed genome.

**GenomeSPOT growth condition predictions**. This section reports genome-derived predictions for oxygen tolerance, pH range, salinity range and temperature range. These outputs are useful for hypothesis generation and for comparing computational predictions with known or experimentally measured growth properties. They should not be interpreted as direct phenotypic measurements.

**gutSMASH metabolic clusters**. This section identifies gut microbiome-relevant metabolic regions. It reports the predicted region type, compound or pathway class, genomic coordinates, the most similar known cluster and similarity score. This module complements antiSMASH by focusing on primary and specialized metabolic functions relevant to gut-associated microbial metabolism.

**GO enrichment**. This section reports enriched GO terms with fold enrichment and FDR values. It provides a statistical functional enrichment layer based on GO annotations and highlights overrepresented biological processes, molecular functions or cellular components. Enriched terms may include vitamin metabolism, carbohydrate metabolism, nucleotide metabolism, stress response, amino acid biosynthesis, cell wall processes and other functional themes relevant to probiotic genome interpretation.

**Genome GO functional landscape**. This section visualizes GO term distribution across the genome. Bubble size represents gene count, position represents genome coverage and color indicates GO branch. The visualization gives a broad overview of how GO-annotated functions are distributed and represented across the annotated genome.

**Probiotic GO categories**. This section condenses GO evidence into probiotic-relevant categories. It combines fold enrichment, PGMDB-supported genes and evidence tiers to provide a higher-level interpretation of GO results in the context of probiotic traits. This section links general functional annotation with the custom probiotic interpretation layer.

**Results directory structure**. The final section documents the main output files generated by each tool and module. It lists the expected directory paths for quality-control files, assemblies, annotation files, AMR and virulence tables, plasmid and mobile element outputs, functional annotation files, pathway summaries, probiotic marker tables, GO visualizations, genome maps and final reports. This section improves transparency and allows users to locate the underlying files behind each report page.

## 4. Availability and Installation

PROBEAT is available from the GitHub repository https://github.com/vebaev/PROBEAT, accessed on 1 August 2026. The repository contains the Snakemake workflow, Docker and Docker Compose configuration files, Streamlit-based graphical interface, Redis/worker execution setup, database initialization utilities, custom probiotic-oriented databases, reporting scripts and example configuration files. Full installation instructions, including all required commands for building the image, automated database downloading and starting the workflow, are provided in the repository. Most tools have integrated DB downloader which is used by the workflow. DBs which the script downloads are also indicated at https://github.com/vebaev/PROBEAT/tree/master/data/databases, accessed on 1 August 2026.

PROBEAT is distributed as a Dockerized workflow to simplify installation and improve reproducibility across computational environments. The recommended deployment environment is a Linux workstation or Linux server with Docker Engine and Docker Compose installed. Sufficient disk space should be reserved for the Docker image, workflow outputs and downloaded databases; a full installation requires approximately 200 GB of available storage (including the databases of the tools). A Linux machine is recommended to have 16 CPU cores and at least 32 GB RAM. Execution time depends on the input data type, sequencing depth, resulting genome size and computational resources, but a typical bacterial genome analysis may require approximately 1–2 h under the default resource settings.

## 5. Conclusions: Strengths and Limitations of the Workflow

The main advantages of PROBEAT workflow are not only the integration of the well-established analysis tools into single workflow reporting integrated output but the addition of a dedicated probiotic focused layer, including the curated Probiotic Gene Markers database, on top of standard bacterial WGS analysis. This reduces the need for manual tool execution and marker-gene table curation and can support a more standardized comparison of candidate probiotic isolates.

PROBEAT should not be considered a definitive or stand-alone solution for probiotic validation, but rather a complementary software framework that integrates many established analysis tools and simplifies the generation of a unified, interpretable report. Several limitations should be considered when interpreting PROBEAT outputs. First, PROBEAT provides genome-based predictions and functional annotations, but it does not experimentally confirm probiotic activity, antimicrobial activity, safety, or host effects. Therefore, detected markers should be interpreted as genomic potential and require validation through appropriate in vitro, in vivo or clinical assays. Second, the PGMDB has dependence on currently available literature knowledge and therefore may not detect novel, poorly characterized, or highly divergent probiotic-associated genes. The results depend also on the quality of the input reads, genome assembly and the completeness of the external databases integrated with the tools. Future PROBEAT releases will address these limitations through the integration of additional state-of-the-art analysis tools that improve the workflow. PGMDB will also be continuously expanded and updated with newly reported probiotic-associated genes, functional categories, and supporting evidence from scientific literature.

By bringing the major stages of probiogenomic analysis into a single reproducible framework, the workflow reduces the fragmentation and technical complexity associated with conventional multi-tool approaches. PROBEAT integrates read processing, genome assembly, quality assessment, taxonomic classification, annotation, safety screening, functional profiling, metabolic analysis, and probiotic-associated marker detection. The graphical interface, standardized outputs, and automated PDF booklet-style report improve accessibility, traceability, and consistency across studies. Combining curated probiotic-associated genes with established bacterial genomics tools enables simultaneous integration of general genome quality and potential functional features relevant to probiotic potential. Although the workflow does not replace phenotypic, safety, or clinical validation, it provides a structured evidence base for strain characterization, comparative analysis, and experimental design insights. This integrated approach can support more efficient, transparent, and standardized characterization of candidate probiotic bacterial isolates.

## Figures and Tables

**Table 1:** Summary table of the tools used in PROBEAT workflow.

Workflow Tool	Purpose	Output
fastp 1.3.3	Read trimming	FASTQ QC
Porechop 0.2.4	Adapter removal	Trimmed FASTQ
Rasusa 2.1.0	Coverage normalization	Subsampled FASTQ
Unicycler 0.5.1	Short/hybrid assembly	Assembly FASTA
Flye 2.9.6	ONT assembly	Assembly FASTA
Medaka 2.x	Consensus polishing	Polished FASTA
QUAST 5.3.0	Assembly statistics	Quality table
BUSCO 6.0.0	Completeness assessment	BUSCO summary
Mash 2.3; sourmash 4.9	Reference matching	Top-hit tables
FastANI 1.34	ANI comparison	ANI table/heatmap
Bakta 1.12.0	Feature annotation	GFF3/FAA/TSV
eggNOG-mapper 2.1.x	Function assignment	Annotation table
KOfamScan 1.3.0	KO assignment	KO table
KEGGDecoder 1.3	Pathway completeness	Table/SVG
dbCAN 5.2.9	Carbohydrate profiling	CAZyme/CGC tables
AMRFinderPlus 4.2.7	Resistance detection	AMR table
ABRicate 1.0.1	Gene screening	Hit tables
MOB-suite 3.1.9	Plasmid classification	Contig report
geNomad 1.x	MGE detection	Plasmid/virus tables
BAGEL4 1.2	Bacteriocin detection	Cluster table
antiSMASH 8.0.4	BGC detection	region table
gutSMASH 2.0.1	Cluster detection	region table
GenomeSPOT 1.0.1	Condition prediction	Prediction table
PGMDB 1.0	Marker detection	marker summary hits
GOProbiotic 1.0.0	Functional profiling	GO tables/plots
pyCirclize 1.10.1	Circular mapping	PNG/SVG map
MultiQC 1.35	Report aggregation	HTML report

## Data Availability

The repository and the data of the software can be accessed at https://github.com/vebaev/PROBEAT, accessed on 1 August 2026.
